# Risk Factors Associated With Health Care Utilization in Preschool Recurrent Wheezers in a Tropical Environment

**DOI:** 10.3389/falgy.2021.761492

**Published:** 2021-10-28

**Authors:** César Muñoz, Lissette Guevara, María-Isabel Escamilla, Ronald Regino, Nathalie Acevedo, Jose Miguel Escamilla-Arrieta

**Affiliations:** ^1^Department of Pediatrics, University of Cartagena and Pediatric Pulmonology Service, Hospital Infantil Napoleón Franco Pareja–La Casa del Niño, Cartagena, Colombia; ^2^Department of Pediatrics and Sleep Medicine, Fundación Neumológica Colombiana, Bogotá, Colombia; ^3^Institute for Immunological Research, University of Cartagena, Cartagena, Colombia

**Keywords:** atopy, childhood, emergency room visit, wheezing, preschool, tropic

## Abstract

**Introduction:** The severity of wheezing episodes is related with the need for health services, but the factors associated with health care utilization in preschool recurrent wheezers in underdeveloped regions are unclear.

**Objective:** To evaluate the factors associated with health care utilization in preschool recurrent wheezers in Cartagena, Colombia.

**Methods:** One hundred twenty-seven recurrent wheezers (age 2–6 years old) who were admitted to the emergency room (ER) due to wheezing in a Pediatric reference hospital in Cartagena were included. Children were evaluated by means of questionnaires and classified according to the number of ER visits, need for hospitalization and history of intensive care unit (ICU) admission due to wheezing within the last year. Total serum IgE and specific IgE to house dust mite allergens (HDM) were measured by ImmunoCAP® and allergen sensitization was evaluated by skin prick tests (SPT).

**Results:** The maternal report of nocturnal cough without fever in their children increased the risk to have ≥5 ER visits in the last year due to wheezing. The use of montelukast was negatively associated with hospitalization, while a history of pneumonia and lack of tap water, increased the risk of hospitalization due to wheezing. A history of bronchiolitis, family history of asthma, cohabiting with two or more siblings, passive exposure to smoke and lack of sewage facilities increased the risk of ICU admission due to wheezing. The presence of atopy evaluated by SPT reactivity, total IgE levels or specific IgE to HDM were not associated with health care utilization. We also found that seroprevalence of positive IgE (≥0.35 kU/L) was 27% to *B. tropicalis* and 20.3% to *D. pteronyssinus* but the prevalence of positive IgE sensitization to these allergens was below 2% and 8% when evaluated by SPT, respectively.

**Conclusions:** Poverty indicators are associated with ICU admission in a group of preschool recurrent wheezers and should be considered as aggravating factors for wheezing. These factors must be systematically assessed in the medical approach in underdeveloped regions in the tropics. Nocturnal cough without fever is a symptom associated with frequent ER visits while atopy was not associated with health care utilization in preschool recurrent wheezers.

## Introduction

Wheezing is one of the most common respiratory symptoms in preschool children, and it has a myriad of causes and various management strategies. About 40% of children have at least one wheezing episode before the age 6 years (1–4), and although most will overcome them over time without consequences, about one third of wheezers will develop asthma later in life (3, 4). Several studies conducted in temperate/industrialized regions classify wheezing into several phenotypes, but few patients can specifically be assigned to one of them (5–9). Unfortunately, these classifications are not useful for identifying the severity or for guiding management (6, 10).

There is no consensus on the definition of wheezing severity in preschool children. As an attempt to define severity in this age group, the Spanish Guideline for Asthma Management (GEMA, for its Spanish initials) makes an important statement about the nature of asthma in infants, suggesting that it is an episodic disease with asymptomatic periods between crises, and for that reason, other classifications based on adult asthma cannot be applied in children (11). In accordance with GEMA, the Japanese guideline of childhood asthma uses the frequency of symptoms to classify asthma severity in preschool children (and the need of controller medication) compared to adults (12). Other guidelines, such as the Expert Panel Report 3 (EPR3) from the National Heart, Lung, and Blood Institute, have used the need for hospitalization, ICU admission (13), and use of oral/parenteral corticosteroids over the previous year (14) as proxies for the risk aspect of severity.

It has been recognized that in underdeveloped tropical regions, the wheezing prevalence can be similar or even higher than those reported in developed, industrialized regions (15–19). This predisposes the population to a high level of morbidity, especially in preschool children (20). In Colombia, the prevalence of asthma in the general population is 12%. In children below 4 years of age, it reaches 19% (21). In this age group, the clinical patterns of wheezing episodes are typically exacerbated by acute respiratory infections. The severity of these episodes is presumed to be higher compared to those in temperate industrialized settings (22) possibly due to limited access to appropriate treatment and exposure to noxious environments in underdeveloped urban areas (16, 23). This is reflected in an increased number of ER visits, hospitalizations, and ICU admissions.

Some studies have analyzed the environmental risk factors associated with recurrent wheezing in populations of underdeveloped tropical regions (16, 24–26) but few have analyzed the relationship between environmental factors and health care utilization (27). A prospective birth study in an underdeveloped urban community from Cartagena reported a prevalence of recurrent wheezing of 14.2% during the first 2 years of life (24). In the present study, we aim to identify factors associated with health care utilization in preschool children with recurrent wheezing in Cartagena, Colombia.

## Methods

### Study Population

**Figure 1:**
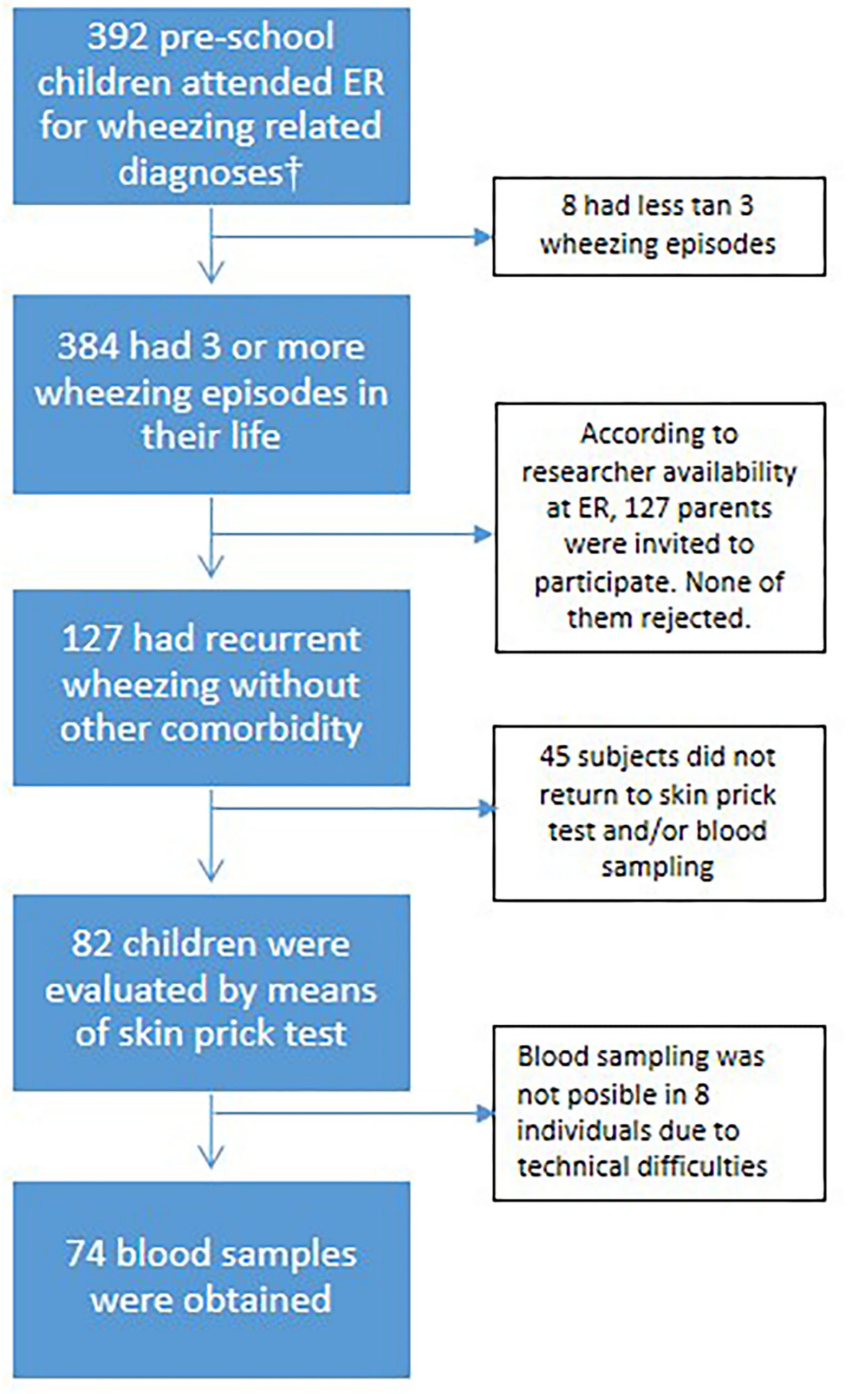
Flowchart of participant's selection process. †The admission codes according to the International Statistical Classification of Diseases and Related Health Problems (ICD-10) were: J40X, J42X, J441, J450, J451, J458, J459, J46X, and J219.

A cross-sectional study was designed. One hundred twenty-seven children, between 2 and 6 years old, were recruited while attending the ER department at Hospital Infantil Napoleon Franco Pareja, a third level, pediatric reference hospital in Cartagena, Colombia. For eligibility, individuals must have had a history of at least three broncho-obstructive episodes in their life and had to experience a physician-confirmed wheezing episode that was improved with a short-acting bronchodilator during their ER visit (Figure 1). Children with other diagnoses or comorbidities that impaired lung function (cystic fibrosis, broncho-pulmonary dysplasia, airway malformations, or cardiac or neurologic abnormalities) were excluded. Questionnaires assessing demographic and environmental risk factors were administered by a trained physician to the accompanying parent, together with a clinical history and a physical examination. All the children were invited to return 2 weeks after discharge from the ER or hospitalization for blood sampling and SPT. This study was approved by the ethical committee of the Hospital Infantil Napoleon Franco Pareja (Act. 8-16/03/8), the parents provided written informed consent to participate for all the children and patient anonymity was preserved.

### Definitions for Health Care Utilization

Three indicators were considered to evaluate health care utilization during wheezing episodes in this population: the number of ER visits during the previous year due to wheezing, a history of hospitalization due to wheezing during the previous year, and a history of admission to an ICU due to wheezing. Considering the sample size, we classified the frequency of ER visits into <5 and ≥5 episodes, so the groups “frequent episodes” and “persistent wheezers” according to GEMA (11), were merged here into one group (*n* = 42) and analyzed in all subsequent analyses. These indicators were reported by the parents when interrogated using questionnaires at the ER.

### Sociodemographic and Clinical Indicators

Previous validated questionnaires (24) were used to document sociodemographic conditions (age, gender, strata, housing conditions, siblings, pets, environmental exposures), details of the wheezing phenotype (age of onset, number of ER visits, hospitalizations, and ICU admissions, and use of medications), personal history of allergic diseases (asthma, rhinitis or eczema), family history of allergic diseases, perinatal conditions, and personal history of infectious diseases. Socioeconomic strata in Cartagena are classified from 1 to 6, according to poverty indicators assessed by the municipality. Overcrowding was defined in accordance with the World Health Organization as 2.5 or more people sleeping in the same room. Since the definition of overcrowding varies throughout the literature and it is typically defined as the ratio of people per room in a house (WHO Housing and health guidelines. Geneva: World Health Organization; 2018). We here defined overcrowding as 2.5 or more people per room following the guide of the Economic Commission for Latin America and the Caribbean CELADE (http://hdl.handle.net/11362/9781). Children under 1 year of age were not included in this count, but older ones were.

### Allergic Phenotypes

The personal history of allergic diseases was based on a self-report of physician-diagnosed asthma, physician-diagnosed rhinitis, and physician-diagnosed eczema. However, the questionnaire also contained questions for identifying symptoms at the time of recruitment, based on those we defined to be current asthma as three or more lifetime broncho-obstructive episodes and at least one episode during the previous year; current rhinitis was defined as two or more symptoms (rhinorrhea, nasal obstruction, nasal itching, and/or sneezing) described by the ARIA (Allergic Rhinitis and its Impact on Asthma) guidelines, with their severity classified as intermittent, persistent, mild, or moderately severe (28). Eczema was defined according to the criteria of the UK working party (29).

### Skin Prick Tests and IgE Measurements

A subgroup of 82 children returned 2 weeks after discharge from the ER for skin prick tests. Eighteen allergens were tested: *Dermatophagoides farinae* (m602), *Dermatophagoides pteronyssinus* (m601), *Blomia tropicalis* (m608), feather mix (me01), *Aspergillus fumigatus* (p902), wheat (f004), flower mix (mb58), dog (e802), egg yolk (f075), egg albumin (f001), *Alternaria alternata* (p901), cat (e801), soy (f014), milk (f002), *Periplaneta americana* (i703), peanut (f013), grass (mw57), and pork (f026) (Inmunotek, Madrid, Spain). Histamine phosphate (k200) was used as the positive control and glycerol (k100) as the negative control. A test was considered positive when there was a wheal diameter equal or above 3 mm than the negative control after 20 min. Blood samples were taken via antecubital phlebotomy in 74 children. The tubes were incubated at room temperature for 2 h, and the serum was collected after centrifugation at 3000 rpm for 10 min. The serum was stored at −20°C until use. Total IgE and sIgE levels to *Blomia tropicalis* (d201) and *Dermatophagoides pteronyssinus* (d1) were measured by means of ImmunoCAP® (Thermo Fisher, Uppsala, Sweden) at the Institute for Immunological Research of the University of Cartagena. Total IgE levels were reported in IU/ml. sIgE levels above 0.35 kU/L were considered positive for IgE sensitization.

### Statistics

Proportions between groups were compared by the chi square and Fisher exact tests, depending on the number of observations. Since the IgE was non-normally distributed, comparisons with continuous variables were performed with the Mann-Whitney U or Kruskall Wallis tests, as appropriate. Correlations were calculated by means of the Spearman test. In an exploratory manner to identify potential factors associated with health care utilization, the Odds Ratio (OR) was calculated for those variables with different proportions (*p* < 0.05) for ER visits, hospitalization and ICU admission. Those variables were included in a logistic regression model for each outcome, adjusting for the effect of age and gender. A *p*-value below 0.05 was considered statistically significant. Statistical calculations were performed with IBM SPSS Statistics 20 and GraphPad Prism 5.0. The Euler diagram was plotted using the Euler package version 4.1.0 by Larsson, J. (2018) (https://cran.r-project.org/package=eulerr).

## Results

The demographic, clinical characteristics, and environmental exposures of all children are presented in Table 1. Most subjects belonged to the lowest socioeconomic strata in the city and had an onset of wheezing before 12 months. Sixty-seven percent of the children were classified as occasional episodic wheezers, according to GEMA (0–4 episodes/year). About half of them had been hospitalized at least once due to wheezing during the previous year, and 19.7% had been admitted to ICU during their lifetime due to wheezing. A previous physician's diagnosis of asthma was already determined in 68% of the participants, and about 40% were under treatment with beclomethasone. Current rhinitis symptoms were reported by half of the patients, although a physician's diagnosis of rhinitis was only reported in 16.5%. The distribution of risk factors and exposures according to the number of ER visits, hospitalization and ICU admission is presented in Table 2.

**Table 1:** Demographic, clinical characteristics, and environmental exposures of the study children (*n* = 127).

Age (months)	36 (30–48)
Gender (Male)	72 (56.7%)
Lowest socioeconomic strata (strata 1)^a^	75 (59.1%)
**Health services utilization due to wheezing**	
Number of ER visits during the previous year due to wheezing	4 (2–6)
Prevalence of the wheezing phenotype according to the GEMA guideline	
Occasional episodic (0–4 episodes/year)	85 (66.9%)
Frequent episodic (5–8 episodes/year)	24 (18.9%)
Persistent (>8 episodes/year)	18 (14.2%)
History of hospitalization during the previous year	64 (54%)
History of ICU admission during their lifetime	25 (19.7%)
**Allergic manifestations**	
Age at first wheezing episode (months)	7 (3–12)
Report of nocturnal cough without fever	76 (59.8%)
Report of cough while doing sports or exercise	17 (13.4%)
Physician-diagnosed asthma	87 (68.5%)
Current use of beclomethasone	51 (40.2%)
Current use of montelukast	39 (30.7%)
Current rhinitis	64 (50.4%)
Rhinitis phenotypes according to the ARIA guideline (*n* = 64)	
Persistent rhinitis	21 (32.8%)
Moderate to severe rhinitis	32 (50%)
Physician-diagnosed rhinitis	21 (16.5%)
Report of eczema symptoms in the previous 6 months	17 (13.4%)
Any positive SPT (*n* = 82)	24 (29%)
Positive SPT to HDM (*n* = 82)	8 (9.7%)
Positive sIgE to HDM (*n* = 74)	23 (31%)
**Family history of allergic diseases**	
Family history of asthma (mother, father, and/or sibling)	79 (62.2%)
Maternal asthma	23 (18.1%)
Paternal asthma	17 (13.4%)
Family history of rhinitis (mother, father, and/or sibling)	70 (55.1%)
**Perinatal factors**	
Preterm birth (<37 weeks of gestational age)	31 (24.4%)
Cesarean delivery	81 (63.8%)
Neonatal ICU stay	20 (15.7%)
**Reported previous infectious diseases**	
Bronchiolitis	54 (42.5%)
Pneumonia	61 (48%)
Pyoderma	18 (14.2%)
Urinary tract infection	25 (19.7%)
Expulsion of roundworm	32 (25.2%)
**Living conditions**	
Two or more siblings	41 (32.3%)
Overcrowding	30 (23.6%)
Passive exposure to smoke	19 (15%)
Trash burning at home	44 (34.6%)
Pet ownership	53 (41.7%)
dog	37 (29.1%)
cat	14 (11.8%)
Lack of tap water	17 (13.4%)
Lack of sewage facilities	35 (27.5%)

*Continuous variables are in median (interquartile range) and categorical variables in percentage*.

*ER, emergency room; GEMA, Spanish Guideline for Asthma Management (GEMA, for its Spanish initials); ICU, intensive care unit; ARIA, Allergic Rhinitis and its Impact on Asthma guidelines; SPT, skin prick test; HDM, house dust mite; sIgE, specific IgE*;

a *Socioeconomic strata in Cartagena are classified from 1 to 6, according to the residential property and defined by the municipality*.

**Table 2:** Distribution of variables according health services utilization in preschool wheezers.

	**ER visits during the previous year**			**Hospitalization during the previous year**			**ICU admission during their lifetime**		
	**≥5 (*n* = 42)**	** <5 (*n* = 85)**	***P***	**Yes (*n* = 64)**	**No (*n* = 63)**	***p***	**Yes (*n* = 25)**	**No (*n* = 102)**	***p***
Age (months)	36 (31–47)	37 (29–52)	0.5	35 (25–48)	39 (35–52)	**0.012**	33 (24–42)	37 (33–50)	**0.027**
Gender (Male)	27 (64.3%)	45 (52.9%)	0.2	36 (56.2%)	36 (57.1%)	0.9	16 (64%)	56 (54.9%)	0.4
Lowest socioeconomic strata	22 (52.4%)	53 (62.4%)	0.2	41 (64.1%)	34 (54%)	0.2	19 (76%)	56 (54.9%)	0.055
**Health services utilization due to wheezing**									
# of ER during the previous year	–	–	–	4 (2.2–6)	4 (2–5)	0.3	4 (2–5.5)	4 (2–6)	0.4
≥5 ER visits during the previous year	–	–	–	23 (35.9%)	19 (30.2%)	0.4	8 (32%)	34 (33.3%)	0.8
Hospitalization during the previous year	23 (54.8%)	41 (48.2%)	0.4	–	–	–	17 (68%)	47 (46.1%)	0.05
ICU admission during their lifetime	8 (19%)	17 (20%)	0.8	17(26.6%)	8 (12.7%)	0.05	–	–	–
**Allergic manifestations**									
Age of first wheezing episode (months)	7 (3.5–12)	7 (3–14)	0.4	7.5 (3.2–12)	7 (3–14)	0.9	6 (1–12)	8 (3.7–12.5)	0.09
Nocturnal cough without fever	34 (81%)	42 (49.4)	**0.001**	40 (62.5%)	36 (57.1%)	0.5	12 (48%)	64 (62.7%)	0.1
Physician-diagnosed asthma	31 (73.8%)	57 (67.1%)	0.4	45 (70.3%)	43 (68.3%)	0.8	21 (84%)	67 (65.7%)	0.7
Current use of beclomethasone	27 (64.3%)	42 (49.4%)	0.1	33 (51.6%)	36 (57.1%)	0.5	8 (32%)	61 (59.8%)	**0.012**
Current use of montelukast	11 (26.2%)	28 (32.9%)	0.4	12 (18.8%)	27 (42.9%)	**0.003**	4 (16%)	35 (34.5%)	0.092
Current rhinitis	25 (59.5%)	39 (45.9%)	0.1	34 (53.1%)	30 (47.6%)	0.5	10 (40%)	54 (52.9%)	0.2
Current persistent rhinitis	11 (26.2%)	10 (11.8%)	**0.04**	13 (20,3%)	8 (12,7%)	0.3	2 (8%)	19 (18,6%)	0.2
Current moderate to severe rhinitis	8 (19%)	24 (28.2%)	0.2	16 (25%)	16 (25.4%)	0.9	5 (20%)	27 (26.5%)	0.5
Physician-diagnosed rhinitis	6 (14.3%)	15 (17.6%)	0.6	6 (9.4%)	15 (23.8%)	**0.029**	2 (8%)	19 (18.6%)	0.2
Report of eczema symptoms in the previous 6 months	5 (11.9%)	12 (14.1%)	0.7	10 (15.6%)	7 (11.1%)	0.4	4 (16%)	13 (12.7%)	0.6
Any positive SPT (*n* = 82)	12 (33.3%)	12 (26.21%)	0.4	12 (36.4%)	12 (24.5%)	0.2	3 (27.3%)	21 (29.6%)	0.8
**Family history of allergic diseases**									
Family history of asthma (mother, father, and/or sibling)	28 (66.7%)	51 (60%)	0.4	37 (57.8%)	42 (66.7%)	0.3	20 (80%)	59 (57.8%)	**0.041**
Family history of rhinitis (mother, father, and/or sibling)	24 (57.1%)	46 (54.1%)	0.7	31 (48.4%)	39 (61.9%)	0.1	7 (28%)	63 (61.8%)	**0.002**
**Perinatal factors**									
Preterm birth (<37 weeks of gestational age)	10 (23.8%)	21 (24.7%)	0.9	14 (21%)	17 (27%)	0.5	4 (16%)	27 (26.5%)	0.2
Cesarean delivery	23 (54.8%)	58 (68.2%)	0.1	41 (64.1%)	40 (63.5%)	0.9	17 (68%)	64 (62.7%)	0.6
Neonatal ICU stay	9 (21.4%)	11 (12.9%)	0.2	13 (20.3%)	7 (11.1%)	0.1	4 (16%)	16 (15.7%)	0.9
**Reported previous infectious diseases**									
Bronchiolitis	22 (52.4%)	32 (37.6%)	0.1	26 (40.6%)	28 (44.4%)	0.6	17 (68%)	37 (36.3%)	**0.004**
Pneumonia	21 (50%)	40 (47.1%)	0.7	37 (57.8%)	24 (38.1%)	**0.026**	16 (64%)	45 (44.1%)	0.075
Expulsion of roundworm	9 (21.4%)	23 (27.1%)	0.4	18 (28.1%)	14 (22.2%)	0.4	10 (40%)	22 (21.6%)	0.057
**Living conditions**									
Two or more siblings	30 (71.4%)	56 (65.9%)	0.5	25 (39.1%)	16 (25.4%)	0.1	15 (60%)	26 (25.5%)	**0.001**
Overcrowding	10 (23.8%)	20 (23.5%)	0.9	11 (17.2%)	19 (30.2%)	0.085	10 (40%)	20 (19.6%)	**0.031**
Passive exposure to smoke	5 (11.9%)	14 (16.5%)	0.6	9 (14.1%)	10 (15.9%)	0.7	8 (32%)	11 (10.8%)	**0.008**
Pet ownership	13 (31%)	40 (47.1%)	0.083	27 (42.2%)	26 (41.3%)	0.9	10 (40%)	43 (42.2%)	0.8
Lack of tap water	2 (4.8%)	15 (17.6%)	0.054	13 (20.3%)	4 (6.3%)	**0.021**	4 (16%)	13 (12.7%)	0.7
Lack of sewage facilities	9 (21.4%)	26 (30.6%)	0.2	22 (34.4%)	13 (20.6%)	0.083	11 (44%)	24 (23.5%)	**0.04**

*Significant P values are highlighted in bold*.

### Factors Associated With the Frequency of ER Visits Due to Wheezing

Report of nocturnal cough without fever (OR 4.3, 95% CI 1.8–10.4, *p* = 0.001) and current persistent rhinitis (OR 2.66, 95% CI 1.02–6.9, *p* = 0.04) were more frequent in children with >5 ER visits in the previous year due to wheezing. Neither other allergic manifestation nor a parental history of asthma or rhinitis were associated with the number of ER visits. Perinatal factors, sociodemographic aspects, living conditions, and history of infectious diseases were not associated with the number of ER visits. Nocturnal cough without fever was the most significant factor associated with increased risk of >5 ER visits during the previous year due to wheezing even after adjustment by age and gender (Table 3).

**Table 3:** Factors associated with more than five ER visits due to wheezing (*n* = 127).

**Variable**	** <5 episodes n (%)**	**≥5 episodes n** **(%)**	**OR (95% CI) crude**	**OR (95% CI) adjusted by age and gender**
Nocturnal cough without fever	41 (48.2)	34 (81)	4.56 (1.8–10.9) *p* = 0.001	4.5 (1.8–10.9) *p* = 0.001
Current persistent rhinitis	10 (11.8)	11 (26.2)	2.6 (1.02–6.9) *p* = 0.04	2.9 (1.1–7.8) *p* = 0.03

### Factors Associated With Hospitalization During the Previous Year Due to Wheezing

Children who were hospitalized were significantly younger than children who did not (median 35 months, IQR 25–48 vs. 39 months, IQR 35–52, *p* = 0.01, respectively). A history of pneumonia (OR 2.22, 95% CI 1.09–4.5, *p* = 0.026) was more frequent in children who were hospitalized during the previous year due to wheezing. Regarding living conditions, lack of tap water was also more frequent in hospitalized children (OR 3.7, 95% CI 1.15–12.2, *p* = 0.028). By contrast, current use of montelukast (OR 0.3, 95% CI 0.13–0.68, *p* < 0.01) and a physician's diagnosis of rhinitis (OR 0.33, 95% CI 0.11–0.91, *p* = 0.02) were more frequently reported among children that have not being hospitalized due to wheezing. Among these variables, current use of montelukast and a history of pneumonia remained significant after adjustment by age and gender (Table 4).

**Table 4:** Factors associated with hospitalization in the previous year due to wheezing (*n* = 127).

**Variable**	**No n (%)**	**Yes** **n (%)**	**OR (95% CI) crude**	**OR (95% CI) adjusted by age and gender**
Use of montelukast	27 (42.9)	12(18.8)	0.30 (0.13–0.68) *p* = 0.004	0.30 (0.13–0.68) *p* = 0.004
Physician-diagnosed rhinitis	15 (23.8)	6 (9.4)	0.33 (0.1–0.9) *p* = 0.034	0.33 (0.11–0.9) *p* = 0.038
History of pneumonia	24 (38.1)	37(57.8)	2.22 (1.09–4.5) *p* = 0.027	2.1 (1.02–4.3) *p* = 0.044
Lack of tap water	4 (6.3)	13 (20.3)	3.7 (1.1–12.2) *p* = 0.028	3.5 (1.05–11.6) *p* = 0.041

### Factors Associated With ICU Admission During Their Lifetime Due to Wheezing

Current use of beclomethasone (OR 0.31, 95% CI 0.12–0.8, *p* = 0.01) and a family history of rhinitis (OR 0.24, 95% CI 0.09–0.62, *p* = 0.004) were less frequent in the ICU admission group. On the other hand, a history of bronchiolitis (OR 3.7, 95% CI 1.4–9.4, *p* = 0.006) or history of maternal asthma (OR 2.9, 95% CI 1.01–8.3, *p* = 0.05) were more frequent in children with ICU admission. Regarding socioeconomic factors, co-habiting with two or more siblings (OR 4.3, 95% CI 1.7–10.9, *p* = 0.002), exposure to passive smoke at home (OR 3.8, 95% CI 1.3–11.09, *p* = 0.01) and lack of sewage facilities (OR 2.5, 95% CI 1.02–6.3, *p* = 0.04) were more prevalent in children with a history of ICU admission due to wheezing. Overcrowding was also associated with ICU admission (OR 2.7, 95% CI 1.07–6.9, *p* = 0.03). Most of these factors remained associated with ICU admission due to wheezing after adjustment by age and gender (Table 5).

**Table 5:** Factors associated with ICU admission due to wheezing (*n* = 127).

**Variable**	**No (*n* = 102)** **n (%)**	**Yes (*n* = 25)** **n (%)**	**OR (95% CI) crude**	**OR (95% CI) adjusted by age and gender**
Current use of beclomethasone	61 (59.8%)	8 (32%)	0.31 (0.12–0.8) *p* = 0.015	0.29 (0.11–0.77) *p* = 0.012
History of bronchiolitis	37 (36.3%)	17 (68%)	3.7 (1.4–9.4) *p* = 0.006	3.2 (1.2–8.5) *p* = 0.015
History of maternal asthma	15 (14.7%)	8 (32%)	2.7 (1.0–7.4) *p* = 0.05	3.42 (1.18–9.9) *p* = 0.023
Family history of rhinitis	63 (61.8%)	7 (28%)	0.24 (0.09–0.62) *p* = 0.004	0.24 (0.09–0.65) *p* = 0.005
Overcrowding	20 (19.6%)	10 (40%)	2.7 (1.07–6.9) *p* = 0.036	2.6 (1.02–6.8) *p* = 0.045
Cohabiting with two or more siblings	26 (25.5%)	15 (60%)	4.3 (1.7–10.9) *p* = 0.002	5.6 (2.1–15.2) *p* = 0.001
Exposure to passive smoke	11 (10.8%)	8 (32%)	3.8 (1.3–11.09) *p* = 0.01	4.8 (1.5–14.7) *p* = 0.006
Lack of sewage facilities	24 (23.5%)	11 (44%)	2.5 (1.02–6.3) *p* = 0.04	2.35 (0.92–5.9) *p* = 0.07
History of parasite expulsion	22 (21.6%)	10 (40%)	2.42 (0.95–6.13) *p* = 0.062	2.5 (0.97–6.5) *p =* 0.057

### IgE Sensitization and Health Care Utilization

**Figure 2:**
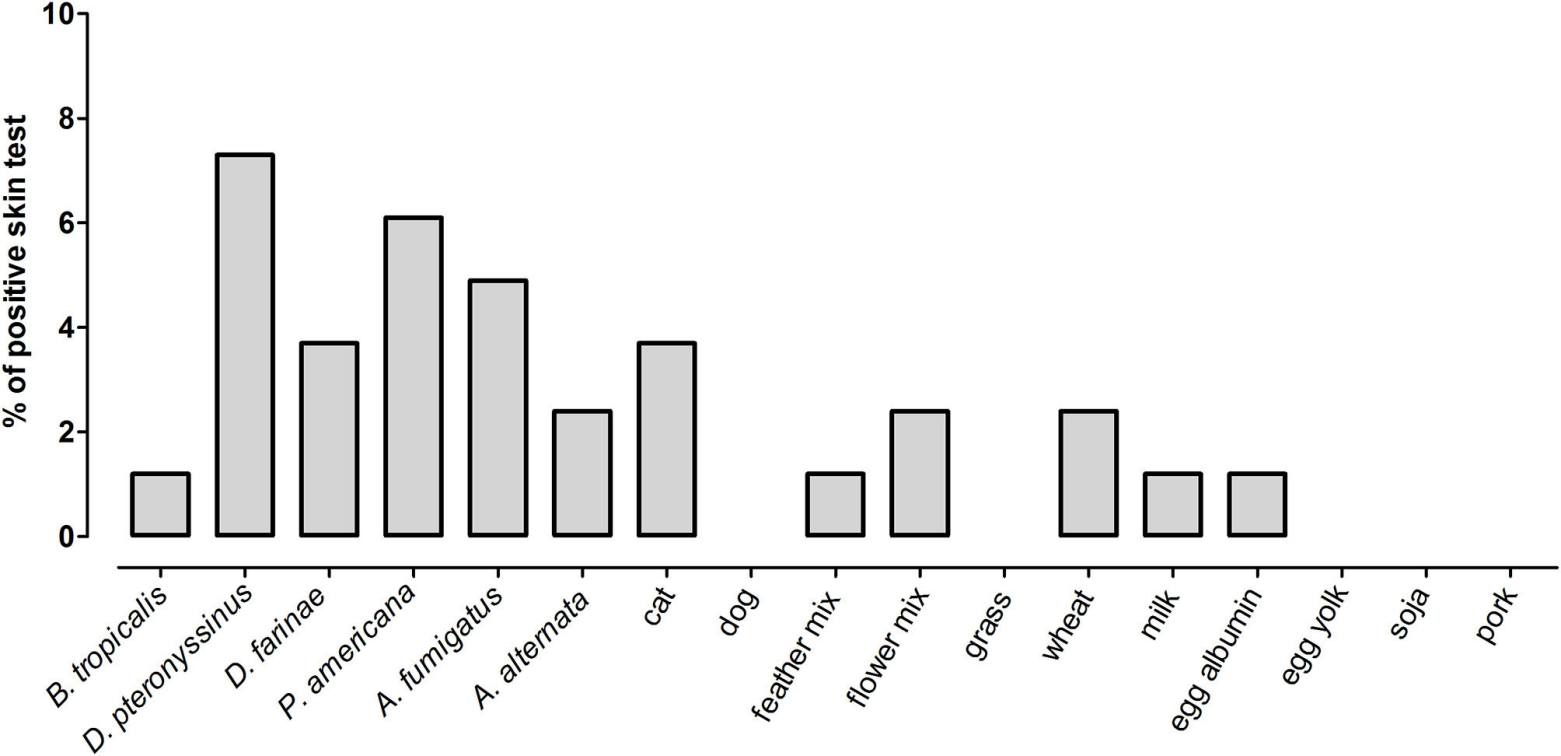
Prevalence of positive skin tests to 18 allergens in a subset of wheezing children that returned 2 weeks after ER discharge (*n* = 82).

We then analyzed the relationship between IgE sensitization to common allergens with the number of ER visits, hospitalization, or ICU admission due to wheezing in 82 children that returned 2 weeks after discharge from the ER (Figure 1). Skin prick tests (SPT) were performed on a battery of 18 allergens. The prevalence of sensitization to any of the allergens tested was 26.8% (*n* = 22). The pattern of sensitization is presented in Figure 2. House dust mite and cockroaches were the most frequent sensitizers in this population (9.7 and 5.6%, respectively). We found no differences in sensitization rates according to the number of ER visits, hospitalization, or ICU admission. Nevertheless, due to the low number of observations per group we cannot rule out a contribution of atopy on health care utilization in recurrent wheezers.

**Figure 3:**
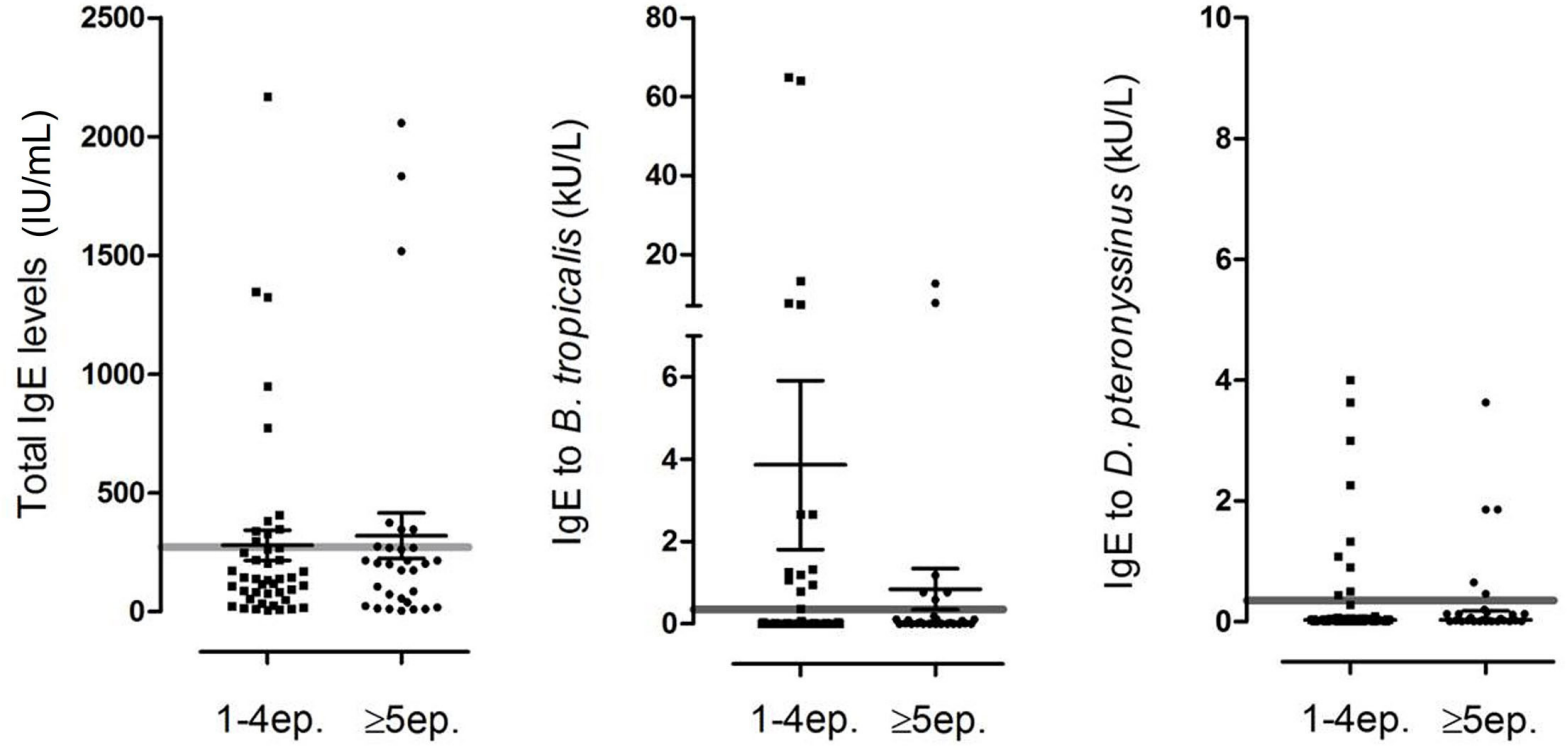
Distribution of total and specific IgE levels in 74 children in which blood tests were available 2 weeks after discharge according to the number of ER visits due to wheezing. Each dot represents an individual, error bars indicate geometric mean and 95% confidence interval. Gray line indicates 300 IU/mL for total IgE and 0.35 kU/L for specific IgE.

**Figure 4:**
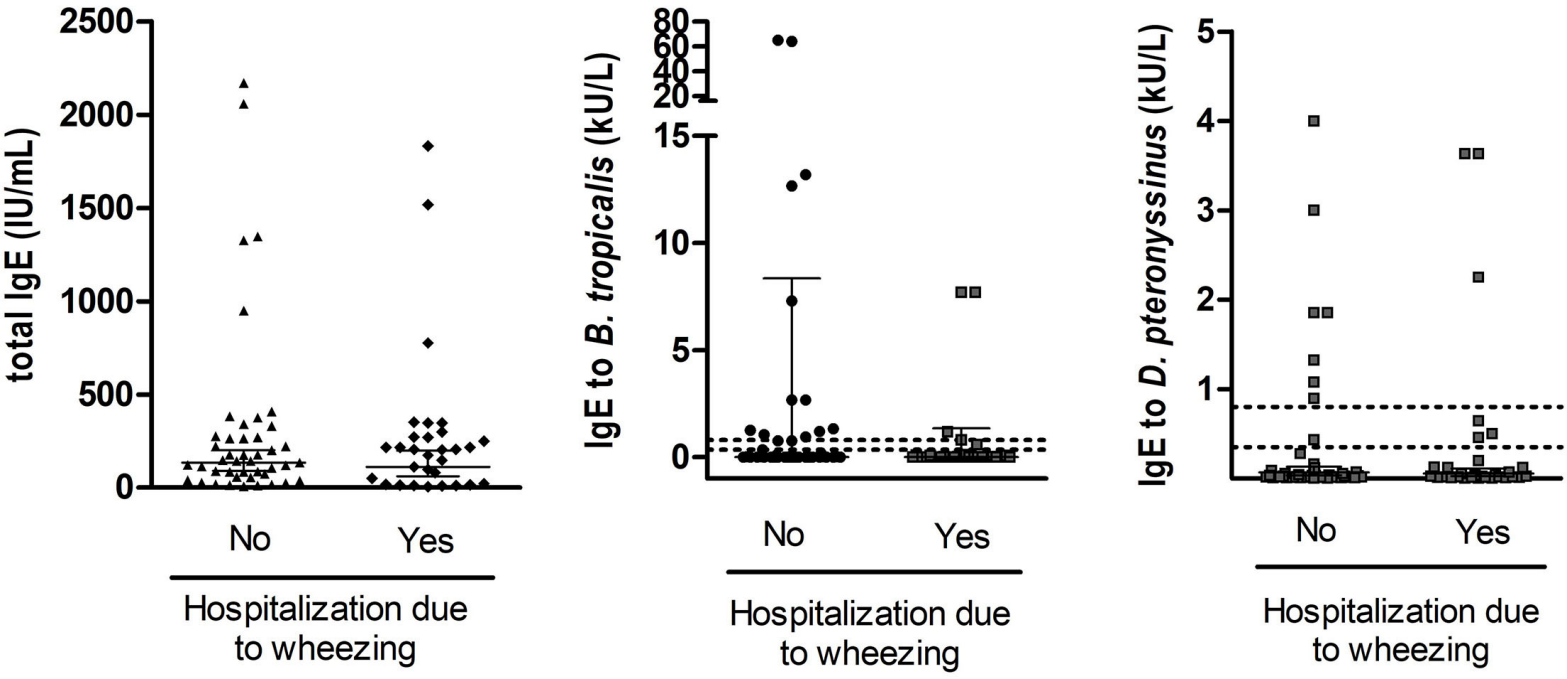
Distribution of total and specific IgE levels in 74 children in which blood tests were available 2 weeks after discharge according to the antecedent of hospitalization in the previous year due to wheezing. Each dot represents an individual, error bars indicate geometric mean and 95% confidence interval. Discontinuous lines indicate the 0.35 and the 0.8 kU/l cut-off for positive IgE sensitization.

We also measured total and specific IgE in 74 children in which a blood sample was obtained. Median levels of total serum IgE were 159 IU/ml (IQR 55–272 IU/ml) and 0.01 kU/L for *B. tropicalis* (IQR 0.002–0.63) and 0.02 kU/L for *D. pteronyssinus* (IQR 0.01–0.13). There was a direct and significant correlation between total IgE levels and the specific IgE levels to *B. tropicalis* (Spearman rho = 0.61, *p* < 0.0001) and *D. pteronyssinus* (Spearman rho = 0.53, *p* < 0.0001). The seroprevalence of positive IgE (≥0.35 kU/L) to *B. tropicalis* was 27% and to *D. pteronyssinus* was 20.3%. Interestingly, the frequency of IgE reactivity to these allergens was considerably higher than those observed by SPT (Figure 2). When analyzed as continuous variables, we found no difference in the total or the specific IgE levels according to the number of ER visits, hospitalizations, or ICU admission. The distribution of IgE levels according ER visits and the antecedent of hospitalization due to wheezing is presented in Figures 3, 4, respectively. When stratifying the children with low and high total IgE levels according to the 75th percentile of the distribution we found no association with any of the outcomes. Likewise, a positive IgE result to *B. tropicalis* or *D. pteronyssinus* (>0.35 kU/L) analyzed as a categorical variable was not associated with health care utilization.

## Discussion

This study reveals the potential factors associated with the number of ER visits, hospitalization, and ICU admissions due to wheezing in preschool children in an underdeveloped urban tropical setting, and it is one of the few that reveals particular risk factors for health care utilization due to wheezing in the tropics. The results are representative of those preschool children, in the lowest socioeconomic strata, with recurrent wheezing who seek medical services in Cartagena due to an exacerbation of the disease and agree with previous associations between poverty and poor hygiene with recurrent wheezing (23, 26, 30).

We found an increased prevalence of nocturnal cough and current persistent rhinitis in children with five or more ER visits during the previous year due to wheezing. This association has not been evaluated in previous studies. The nocturnal pattern of asthma symptoms, like dry cough, has been related to multi-triggers preschool wheezers and allergic phenotypes (31, 32). Interestingly, in our study none of the atopic markers (SPT or sIgE) or a parental history of asthma/allergies were associated with the number of ER visits. Current persistent rhinitis in children of this cohort is most probably non-atopic since there was no association of current persistent rhinitis with total IgE levels or sensitization to HDM allergens (data not shown). This clinical variable should be evaluated in a larger sample size to define its association with the recurrent need of ER visits.

The use of montelukast was the most significant protective factor for hospitalization due to wheezing. Some studies in preschool wheezers have shown that montelukast was effective for reducing caregiver-observed wheezing, the need for salbutamol and acute exacerbations that required oral corticosteroids or hospitalization (33, 34). However, a recent meta-analysis did not demonstrate benefit of montelukast in preschoolers with recurrent wheezing, but it raised the need of studies that evaluate a montelukast responder phenotype (35). It is feasible that children with a diagnosis and who were using this medication were more likely to be properly followed up and had access to the health care system or may also reflect awareness of their parents of their respiratory symptoms. On the other hand, a history of pneumonia was a significant factor associated with an increased risk of hospitalization, suggesting that lower respiratory infections may be critical as risk factor in this population. At the same time, some data suggests that pneumonia may be over diagnosed in children with asthma, especially in low-income countries (36, 37).

**Figure 5:**
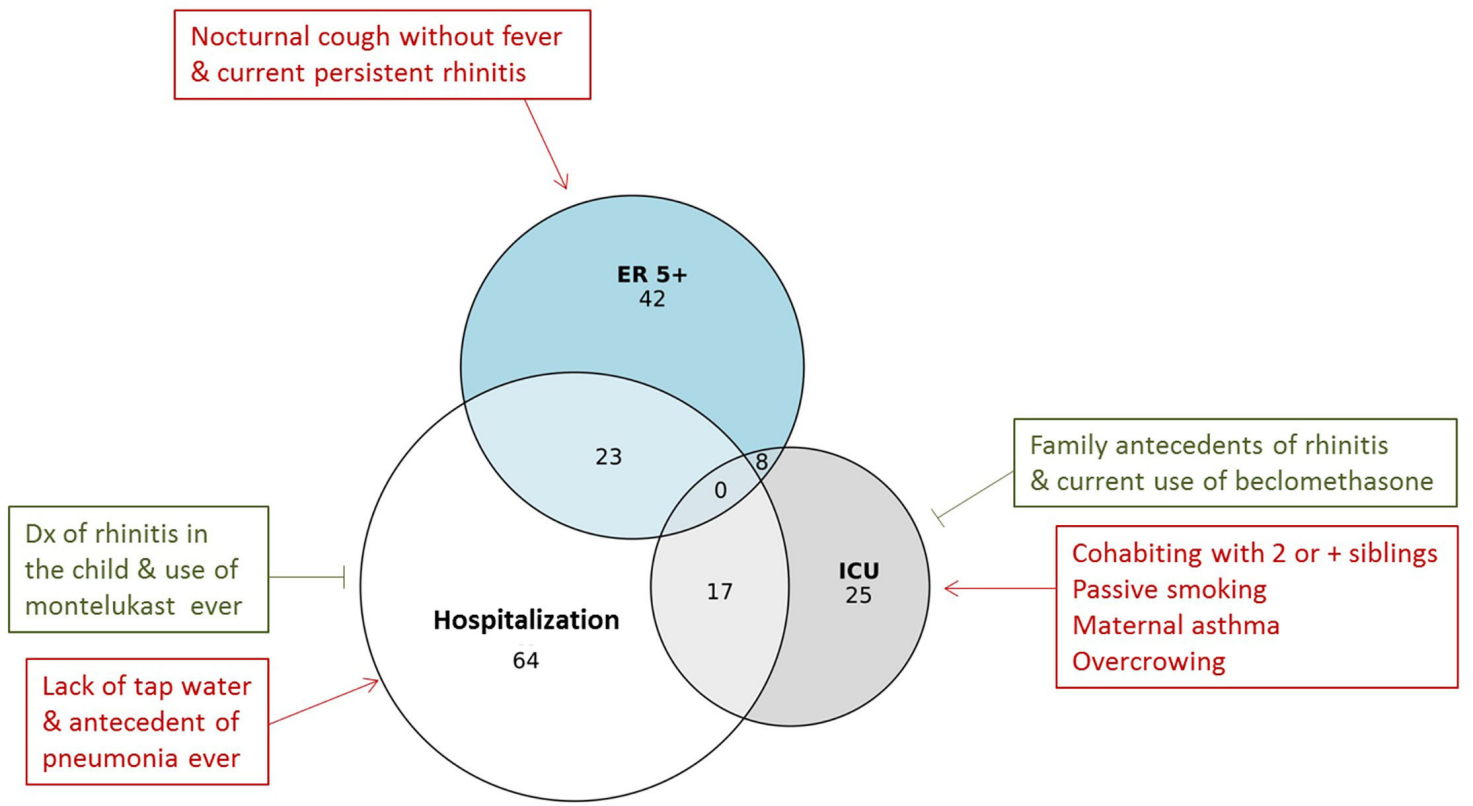
Euler diagram for the overlap among children having had more than 5 ER visits due to wheezing (ER 5+) with those that had been hospitalized and/or admitted to the ICU due to wheezing (*n* = 127). Number of subjects is indicated in the circles. The risk factors and exposures associated to health care utilization are presented within boxes. Red indicates increased risk and green decreased risk.

In this population, 25 children had a history of ICU admission due to wheezing. The most significant factor associated with protection was a family history of rhinitis. These findings could be explained by the fact that people with allergies are more aware of their respiratory symptoms and environmental triggers. Regarding living conditions, cohabiting with two or more siblings, passive exposure to smoke and lack of sewage facilities were the most significant factors associated with the increased risk of ICU admission. While several studies have previously shown that cohabiting with older siblings may protect against further development of allergic asthma, it is quite clear that in urbanized, underdeveloped regions this factor involve the increased number of people per household in the context of overcrowding. This unhygienic environment favors the development of severe communicable infections. Our results are in line with the observation that having older siblings is a risk factor for wheezing (38) and that low income and poverty (poor housing, low birth weight, and parasitic infections) are important risk factors for severe wheezing episodes in young children (39, 40). Further studies are needed to evaluate if the wheezing phenotype in this group reflects a persistent bacterial bronchitis, acute episodes of viral infections, or a different phenotype due to the scarce overlap between the children that had ever required ICU admission and those that had experienced recurrent ER visits (Figure 5). We also detected a non-significant tendency between a family history of asthma and an increased risk of ICU admission. These data support previous observations in a prospective observational study in this population showing that maternal asthma was the most significant factor associated with recurrent wheezing (24). The history of parasite expulsion was associated with increased risk of ICU admission but with marginal significance. This factor could serve as a proxy of the poor living conditions of these children but also could be related with the fact that migration of roundworms can induce pulmonary inflammation (41, 42). Since children with a history of ICU admission were less prone to be analyzed through SPT (44 vs. 69.6% without ICU admission) or serology (32 vs. 64.7% without ICU admission), we could not retrieve a large enough sample size to rule out or confirm involvement of atopy in ICU admission.

Previous studies have found associations between atopy indicators to be important risk factors for predicting asthma at school age (43). However, our results suggest that these factors may not be related to health care utilization for wheezing in this population. For instance, atopy as determined by serology was not associated with any outcome. Indeed, the proportion of preschool children with a positive SPT was low, including allergen sources such as milk and eggs (Figure 2). Further studies are needed to elucidate the biological processes implicated in the differences in prevalence of IgE sensitization when assessed by SPT and serology since this dissociation has been reported (44). A limitation of this study is that we did not evaluate other atopic biomarkers such as eosinophilia. Moreover, we did not analyze the presence of parasitic infection, which could also confound the IgE measurements against HDM extracts due to cross-reactivity (45, 46). Nevertheless, our observations indicate that IgE sensitization to HDM or total IgE was not associated with health care utilization during the wheezing episodes in this age group.

Recall bias is a potential source of confounding in this study, since the only wheezing episode documented by a physician was that by the medical staff at the ER visit. All the other ones were based on parental description. Since previous studies have shown up to 50% discordance between parental-described “wheeze” and physician-documented wheeze (47), here we documented the number of times the children were admitted to the ER due to wheezing, as well as hospitalizations, which are events with a reduced chance of bias due to incorrect parent-defined wheezing. Another limitation of this study is the lack of serology for common viruses or detection of virus in nasal or pharyngeal swabs. This is important for determining if the exacerbations are related to viral wheeze and lower respiratory tract infections (48). Further studies with a larger sample size, new statistical models (49), and appropriate procedures for random sampling and viral detection should be conducted to better define the wheeze phenotypes of these children with recurrent ER visits and those that are more likely to require hospitalizations and/or ICU admissions.

In conclusion, nocturnal cough without fever was the most significant factor associated with increased risk of five or more ER visits during the previous year due to wheezing. On the other hand, children that require ICU admission due to wheezing reported several factors associated with poverty such as cohabiting with two or more siblings, overcrowding, passive exposure to smoke and lack of sewage facilities. Allergic sensitization was not associated with health care utilization although maternal asthma was associated with ICU admission. Moreover, poverty indicators should be considered as aggravating factors for wheezing and could be useful in the development of scores to grade and improve the approach and management of preschool recurrent wheezers from underdeveloped regions in the tropics.

## Data Availability Statement

The raw data supporting the conclusions of this article will be made available by the authors, without undue reservation.

## Ethics Statement

The studies involving human participants were reviewed and approved by the Ethical Committee of the Hospital Infantil Napoleon Franco Pareja (Act. 8-16/03/8), the parents provided written informed consent to participate for all the children and patient anonymity was preserved. Written informed consent to participate in this study was provided by the participants' legal guardian/next of kin.

## Author Contributions

CM and JE-A: conception and design of the study. CM, LG, RR, and JE-A: acquisition of data. CM, NA, and JE-A: analysis and interpretation of data. CM and NA: drafting the article. CM, NA, LG, M-IE, and JE-A: revising it critically for important intellectual content. All authors approved the final version to be submitted.

## Funding

This study was funded by the Asociación Colombiana de Neumología Pediátrica (ACNP) Grant 2015-02 and by the University of Cartagena Resolution 0034/2018. The funders were not involved in preparation of the manuscript.

## Conflict of Interest

The authors declare that the research was conducted in the absence of any commercial or financial relationships that could be construed as a potential conflict of interest.

## Publisher's Note

All claims expressed in this article are solely those of the authors and do not necessarily represent those of their affiliated organizations, or those of the publisher, the editors and the reviewers. Any product that may be evaluated in this article, or claim that may be made by its manufacturer, is not guaranteed or endorsed by the publisher.
